# Can Positive Framing Reduce Nocebo Side Effects? Current Evidence and Recommendation for Future Research

**DOI:** 10.3389/fphar.2019.00167

**Published:** 2019-03-06

**Authors:** Kirsten Barnes, Kate Faasse, Andrew L. Geers, Suzanne G. Helfer, Louise Sharpe, Luana Colloca, Ben Colagiuri

**Affiliations:** ^1^School of Psychology, The University of Sydney, Sydney, NSW, Australia; ^2^School of Psychology, The University of New South Wales, Sydney, NSW, Australia; ^3^Department of Psychology, The University of Toledo, Toledo, OH, United States; ^4^Department of Psychology, Adrian College, Adrian, MI, United States; ^5^Department of Pain and Translational Symptom Science, University of Maryland School of Nursing, Baltimore, MD, United States; ^6^Departments of Anesthesiology and Psychiatry, University of Maryland School of Medicine, Baltimore, MD, United States; ^7^Center to Advance Chronic Pain Research, University of Maryland, Baltimore, MD, United States

**Keywords:** nocebo, placebo, framing, attribute framing, side effects, expectancies, adverse health outcomes, verbal suggestion

## Abstract

Although critical for informed consent, side effect warnings can contribute directly to poorer patient outcomes because they often induce negative expectations that trigger nocebo side effects. Communication strategies that reduce the development of nocebo side effects whilst maintaining informed consent are therefore of considerable interest. We reviewed theoretical and empirical evidence for the use of framing strategies to achieve this. Framing refers to the way in which information about the likelihood or significance of side effects is presented (e.g., negative frame: 30% *will* experience headache vs. positive frame: 70% will *not* experience headache), with the rationale that positively framing such information could diminish nocebo side effects. Relatively few empirical studies (*k* = 6) have tested whether framing strategies can reduce nocebo side effects. Of these, four used attribute framing and two message framing. All but one of the studies found a significant framing effect on at least one aspect of side effects (e.g., experience, attribution, threat), suggesting that framing is a promising strategy for reducing nocebo effects. However, our review also revealed some important open questions regarding these types of framing effects, including, the best method of communicating side effects (written, oral, pictorial), optimal statistical presentation (e.g., frequencies vs. percentages), whether framing affects perceived absolute risk of side effects, and what psychological mechanisms underlie framing effects. Future research that addresses these open questions will be vital for understanding the circumstances in which framing are most likely to be effective.

## Overview

As one participant in a recent study aptly remarked, “If I see all the side effects of the drug I am already ill” (Herber et al., 2014, p. 4). Numerous studies indicate that negative health information can generate negative expectancies that lead to adverse outcomes – labeled the nocebo effect (Colagiuri and Zachariae, 2010; Colloca and Miller, 2011; Faasse and Petrie, 2013). This creates an ethical paradox: informed consent requires that patients are warned about potential side effects (Wells and Kaptchuk, 2012; Colloca, 2015, 2017), but these warnings themselves may produce poorer health outcomes via the nocebo effect (e.g., Myers et al., 1987; Mondaini et al., 2007; Neukirch and Colagiuri, 2015).

The burden of nocebo effects on the healthcare system is not trivial. Nocebo effects account for between 40 and 100% of drug side effects (Mahr et al., 2017). Nocebo-induced side effects can result in treatment termination, protracted treatment, and psychological distress (Barsky et al., 2002). Communication strategies that reduce negative expectancies associated with the nocebo effect, but preserve informed consent, are therefore critical. One strategy that is gaining increasing theoretical attention is framing (e.g., Williams et al., 2013; Colloca and Nestoriuc, 2016; Planès et al., 2016; Glare et al., 2018; Webster et al., 2018a; Petrie and Rief, 2019).

Interest in side effect framing stems from the work of Kahneman and Tversky (Kahneman and Tversky, 1979; Tversky and Kahneman, 1981), who demonstrated that individuals do not appraise information purely rationally and objectively, but are influenced by how that information is presented. In their classic examples (Tversky and Kahneman, 1981), shifts in preference for statistically comparable outcomes were observed when framed in terms of lives saved (positively framed) as opposed to lives lost (negatively framed). Similarly, framing of side effect information to focus on positive outcomes (e.g., likelihood of *not* experiencing side effects) rather than the negative (e.g., likelihood *of* experiencing side effects) may reduce maladaptive expectancies about side effects (Glare et al., 2018), thereby reducing the burden of nocebo side effects (Colloca and Benedetti, 2007; Faasse and Petrie, 2013). A particularly appealing aspect of using framing to reduce nocebo side effects is that because statistical information regarding side effects is equivalent, informed consent is maintained.

**FIGURE 1:**
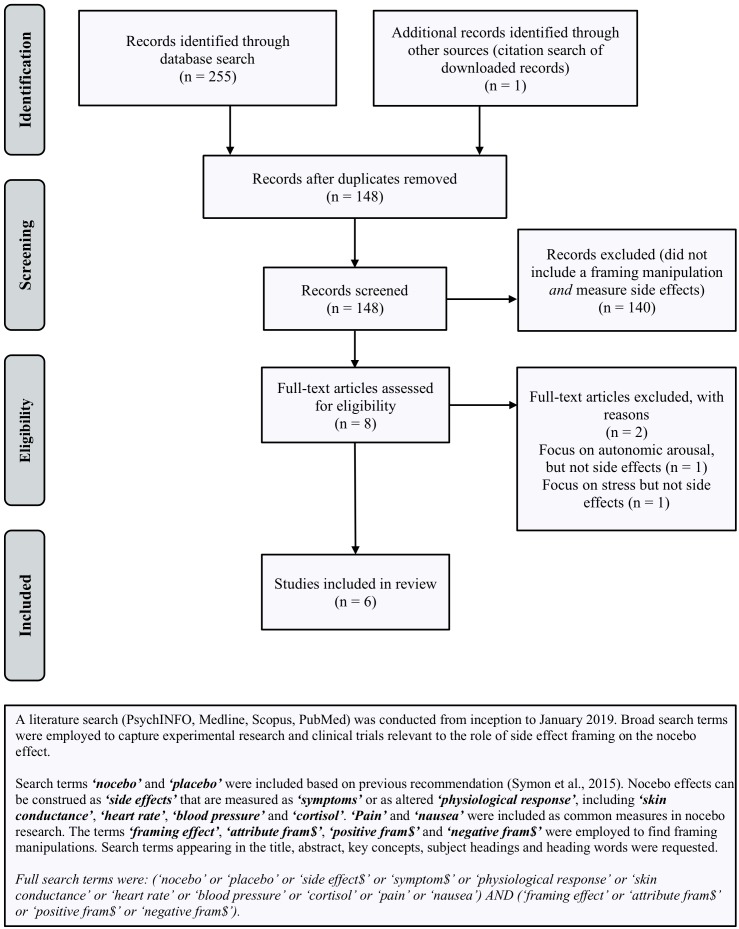
Search terms and PRISMA flow-chart (from: Moher et al., 2009) outlining the procedure used to identify studies included in review.

The effects of framing on health outcomes unrelated to the nocebo effect are well-documented (O’Keefe and Jensen, 2007, 2009; Gallagher and Updegraff, 2012). However, there appears to be surprisingly little empirical research examining whether framing can reduce nocebo-induced side effects and no attempts to synthesize existing studies. To address this, we systematically reviewed studies regarding framing and side effects, in order to identify promising framing strategies for reducing nocebo side effects and make suggestions for future research. Since only a small number of studies were identified, we present the results in narrative fashion. Full details of the search (Figure 1) and methods (Supplementary Material) are provided.

## Evidence to Date

### What Constitutes Side Effect Framing?

Levin et al. (1998) distinguished between three sub-types of framed information: attribute framing (valence framing of a characteristic), risky choice framing (framing of risk information), and goal framing (framing the goal of an action). Due to differentially framing a single characteristic, attribute framing is likely of greatest theoretical relevance to the nocebo effect. The single characteristic is the likelihood of experiencing the symptom, framed positively (will *not*) or negatively (*will*). However, Levin et al. (1998) taxonomy precludes framing types with qualitative differences in the information presented (e.g., “message framing” below). Therefore, we opted for a broader definition and considered any manipulation in which framing was used to accentuate side effects positively, which we refer to as “positive valence framing.” In all, we identified six empirical studies comparing a positive valence frame with another type of frame. Table 1 summarizes these studies.

**Table 1:** Summary of studies included in review.

	Study					
	Fernandez et al. (2019)	Faasse et al. (2018a)	O’Connor et al. (1996)	Webster et al. (2018b)	Wilhelm et al. (2018)	Caplandies et al. (2017)
Participants	Healthy volunteers	Healthy volunteers	Patients with respiratory and cardiac disease	Healthy volunteers	Healthy volunteers	Healthy volunteers
Sample size per group	Positive Frame: 33 Negative Frame: 33	Positive Frame: 36 Negative Frame: 39 Control: 37	Positive Frame: 148 Negative Frame: 144	Positive Frame: 102 Negative Frame: 101	Positive Frame: 40 Negative Frame: 40	Positive Frame: 48 Negative Frame: 51 Control: 27
Treatment	Active treatment 100 g Diclofenac / 1.2 mg Atropine	Placebo tablet described as benzodiazepine for anxiety	Active influenza vaccination	Sham treatment described as a “well known tablet”	Active treatment (beta-blocker) 100 g Metoprolol	Sham brain stimulation (tDCS) to assess cognitive performance
Framing Type	Message Framing	Attribute	Attribute	Attribute (with caveat)^a^	Message Framing	Attribute
Communication method	Video only	Verbal, written, and pictorial	Verbal, written, and pictorial	Written only	Verbal only	Verbal only
Statistical Information	None (verbal descriptor: “frequent”)	Natural frequency	Percentage and natural frequency	Percentage and natural frequency	Natural frequency	Percentage
No. of framed side effects	8^b^	4	5	14	1^c^	1
Outcome Measure	GASE^d^	Modified GASE^d^	Study specific^d^	Modified GASE^d^	Modified GASE^d^	Study specific^d^
Measured Symptoms: occurring generally vs. specifically attributed to treatment	Attributed to treatment	Generally occurring	Generally occurring	Attributed to treatment	Attributed to treatment	Generally occurring
Raw effect size data presented in the paper	Side effect frequency increased by 0.8 symptoms (on a 36 item GASE) in the positive frame	Side effects reduced by 1.42 points on a 11-point GASE	Approximately 17–19% reduction in reporting of myalgia and chills and an 8% drop in work absenteeism	Positive framing group 34% less symptoms attributed to the tablet	Reduction in the intensity of the framed side effect (dizziness) of 0.12 points (4-point GASE) associated with the positive frame	Positive framing group reported headache on 0.34 fewer recording periods (out of 5) compared to negative frame
Descriptive Statistics (framed side effects: *M*; *SD*)	Positive Frame (1.70; 1.44) / Negative Frame (0.91; 0.84)	Positive Frame (2.46; 2.88) / Negative Frame (3.88; 2.94)	Descriptive statistics not published: averaged effect size *r* is reported from the 3 χ^2^ values available (i.e., 8.9, 6.2, 4.3)	Number experiencing side effects (*OR* = 0.66): Positive Frame (*n* = 33; 32.4%) / Negative Frame (*n* = 47; 46.5%)	Positive Frame (0.48; 0.68) / Negative Frame (0.60; 0.63)	Positive Frame (1.28; 1.77) / Negative Frame (1.62; 1.97) / Control (0.48; 1.25)
Largest effect size for framed side effects (Pearson’s *r*)	0.32^e^	0.24^e^	0.19^e^	0.11^e^	0.09^f^	0.09^e^
No. of non-framed side effects assessed	None	22	9	9	28	None
Largest effect size for non-framed side effects (Pearson’s *r*)	N/A	0.15	Not reported	0.06	0.15	N/A
Expectancy measure	Expectancy for relief, not side effects, measured	Single-item, study specific^g^	Six items, study specific^g^	None	None	None
Effect of frame on Expectancy (Pearson’s *r*)	N/A	0.17	0.10^h^	N/A	N/A	N/A
Anxiety Measure	STAI and ASI^i^	STAI^i^	None	STAI Short^i^	None	None
Effect of Anxiety	No difference between groups (STAI *r* = 0.06, *p* = 0.61; ASI *r* = 0.12, *p* = 0.32). ASI associated with more medication attributed side effects (*r* = 0.30, *p* = 0.02)	No overall difference in anxiety between framing groups (*r* = -0.05, *p* = 0.68)	N/A	Increase in anxiety associated with more side effects in negative frame (*r* = 0.02, *p* = 0.04)	N/A	N/A

*^a^ Statistical presentation type and written descriptors differed by frame. ^b^ Eight frequently reported side effects are listed during consent. Wording of the positive frame was “if you experience a side effect, you might take this as a reminder that the analgesic medication is active,” suggesting all side effects are positive (unlike Wilhelm et al., 2018). Side effects are measured via the Generic Assessment of Side Effects Scale (GASE) (see below) and not split into framed and non-framed categories. ^c^ One side effect (dizziness) was framed as positive, four were listed during consent, and eight treatment-specific side effects were statistically analyzed [those considered frequent in the patient information leaflet (PIL)]. As our primary interest is the direct effect of framing on side effects, we focus on side effect intensity for the framed side effect (dizziness). ^d^ GASE (Rief et al., 2011). The GASE is a 36-item scale assessing the experience of symptoms. The scale is rated on a 4-point likert scale (11-point in Faasse et al., 2018a) and can be used to assess intensity of general symptoms as well as frequency and intensity of symptoms attributed to treatment. Headache frequency in Caplandies et al. (2017) was rated on a 5-point scale and embedded among eight other symptoms. Symptom measurement in O’Connor et al. (1996) is based on Scheifele et al. (1990) but not reported in detail in either paper. ^e^ Consistent with other health-related framing reviews (e.g. O’Keefe and Jensen, 2007, 2009; Gallagher and Updegraff, 2012), Pearson’s r was used as a summary effect size. As difference between values are small, effect sizes are used only as a tool to generate discussion. We note that difference in experimental procedures and measured variables can generate extraneous fluctuations in standardized effect sizes (Baguley, 2009). We therefore we urge against strong conclusions. Primary effect sizes are calculated for framed symptoms (i.e., those listed during the consent process). As experiments did not report statistically significant results across all outcome measures, the largest effect size pertaining to either side effect intensity or frequency is listed with description. ^f^ For consistency, we report the effect size for change in side effect intensity of the framed side effect (dizziness) in the case of Wilhelm et al. (2018). We note that the largest effect in this paper is perceived threat of eight drug-specific side-effects (r = 0.18). As only one these side effects was framed, and half not listed during consent, this effect does not form the primary focus of the present article. ^g^ Wording of the expectancy item in Faasse et al. (2018a) was: “how likely are you to experience side effects as a result of taking the benzodiazepine tablet?” (11-point scale). In O’Connor et al. (1996), perceived vaccine risks and benefits (flu, systemic and local side effects) were rated on a 21-point probability scale (0–100%). ^h^ The largest effect size associated with a single item of O’Connor et al.’s (1996) expectancy measure was the expectancy for acquiring local side effects, which differed by 11% between frames (positive 35.6% vs. negative 46.6%). ^i^ The State-Trait Anxiety Inventory (Spielberger, 1983) is a 20-item questionnaire assessing state anxiety. The short version of the scale (Marteau and Bekker, 1992) contains six items. The Anxiety Sensitivity Index (Reiss et al., 1986) is a sixteen-item questionnaire assessing beliefs that the experience of anxiety has negative implications. All measures employ a 4-point scale.*

### Attribute Framing

Four of the six studies identified investigated attribute framing. As above, attribute framing involves identical statistical information being presented either positively (will *not* experience) or negatively (*will* experience). O’Connor et al. (1996) was the first and the only one involving a clinical sample. They compared positive and negative attribute framing regarding influenza vaccines in patients with respiratory and cardiac disease. Patients were provided with verbal, written, and pictorial information regarding side effect risk, presented in a positive (60% will *not* get a sore arm) or negative (40% *will* get a sore arm) attribute frame. Three days post intervention, the positive frame led to fewer reported side effects and less absence from work than the negative frame, indicating a significant framing effect.

Webster et al. (2018b) administered placebo tablets to healthy volunteers described as a well-known medication. Information about multiple possible side effects (e.g., headache, nausea) was framed positively (will *not* be affected) or negatively (*will* be affected) as part of a Patient Information Leaflet (PIL). They implemented a hurdle model (see: Hu et al., 2011) on side effects attributed to the treatment and found that fewer participants reported such side effects following the positive frame. However, number and severity of side effects in those reporting at least one did not differ between frames, suggesting the positive frame primarily affected any occurrence of side effects. Worth also noting, their study does not meet strict criteria for attribute framing (Levin et al., 1998). While side effect risk was framed positively or negatively, statistical presentation type and written descriptors differed by frame (e.g., headache as an “uncommon side effect, 80% *will not* be affected” vs. as a “very common side effect, more than 1 in 10 *will* be affected;” Webster, 2018), which may have influenced results.

Faasse et al. (2018a) administered placebo tablets under the guise of a benzodiazepine and were the only researchers to include a no treatment control. Information about four side effects presented verbally, in writing, and pictorially was either framed positively (will *not* experience) or negatively (*will* experience). Both frames produced nocebo side effects relative to control. The positive frame reduced side effect reporting relative to the negative frame 15 min after treatment, but not 24 h later, suggesting temporary success of the framing manipulation.

Caplandies et al. (2017) were the only researchers to include a no-framing instruction control. They delivered sham brain stimulation to healthy volunteers to induce headaches in a 2 (effect type framing: primary or side effect) × 2 (attribute framing: positive vs. negative) + 1 (no-frame control) design. Participants received a verbal warning about headaches framed either positively (30% *unlikely* to get headache) or negatively (70% *likely* to get a headache), unless assigned to the control, where they received no information regarding side effects. There was an overall nocebo effect relative to control and these nocebo headaches were more likely in primary effect condition compared to side effect condition. However, unlike the above studies, there was no significant attribute framing effect on any measure.

#### Positive Message Framing

Two studies employed positive message framing, involving information that side effects were indicative of the drug working (Wilhelm et al., 2018; Fernandez et al., 2019).

Wilhelm et al. (2018) administered the beta-blocker metoprolol to healthy volunteers and verbally framed dizziness as a positive (indicative of the drug working) or negative occurrence (common unpleasant side effect). Framing had no effect on the frequency or intensity of side effects, but trended toward reducing perceived threat of side effects (Pearson’s *r* = 0.18). Exploratory moderation analysis revealed a reduction in frequency and threat of side effects among a subset of participants (those with high harm beliefs) – but we focus here on the overall sample to ensure consistency across studies.

Fernandez et al. (2019) administered the analgesic diclofenac with a side-effect-inducing agent (atropine) in healthy volunteers. Video instructions stated that experiencing side effects were an indication that the medication was active in the body and would help reduce pain (positive frame) or simply to inform staff if side effects were experienced (control frame). In this case, the positive frame led to more treatment-attributed side effects, but lower overall side effect intensity. Further, in the positive frame, side effect frequency predicted increased analgesia suggesting the positive frame may bolster treatment efficacy via the placebo effect for those who experience side effects.

### Summary

Taken together, three of four studies suggest that positive attribute framing produces small reductions in side effects (effect size range: *r* = 0.11–0.24). In terms of message framing, the results were less consistent, with one study showing mixed effects (increased attribution, but decreased intensity) and the other only showing trends toward reduced side effect threat (i.e., not attribution or intensity).

## Methodological Considerations and Future Directions

While the handful of existing studies suggest that framing is a promising technique for reducing nocebo side effects, they also highlight several unanswered questions that need to be addressed before the widespread use of framing can be recommended.

### What Is the Best Mode of Communication?

The studies reviewed varied in terms of the mode of communication of side effect warnings. Two studies employed written, verbal, and pictorial methods simultaneously (O’Connor et al., 1996; Faasse et al., 2018a), two used verbal (Caplandies et al., 2017; Wilhelm et al., 2018), one video (Fernandez et al., 2019), and one written only (Webster et al., 2018b). The data in Table 1 suggest that the studies using multiple modes (including video, which comprises visual and verbal presentation methods) elicited numerically larger framing effect sizes, suggesting that multi-modal presentation may more successfully elicit the framing effect. Of course, it is impossible to know whether pictorial methods alone or interactions between multiple methods are fundamental to driving the framing effect. Regardless, traditional methods of delivering side effect information, such as in PILs, may be limited for inducing framing effects because they involve a single, written communication mode. Emerging health technologies (Suggs, 2006; Colledge et al., 2008), however, make the multi-media delivery of health information increasingly easy to implement. Thus, future research could capitalize on such techniques and systematically examine the effect of framing in different communication modes. This would help determine the optimal mode of delivery for inducing framing effects and to overcome potentially less efficacious methods (e.g., written PILs).

### What Is the Best Method of Presenting Statistical Information About Side Effects?

Framed information regarding side effect prevalence can be presented in a number of formats, ranging from verbal descriptors (e.g., “common”), natural frequencies (1 in 10) and percentages (10%). Each influences the perception of absolute perceived risk of side effects to varying degrees (e.g., Hoffrage and Gigerenzer, 1998; Yamagishi, 1999; Berry et al., 2003), potentially impacting the nocebo effect. Of the studies reviewed, one employed verbal descriptors (Fernandez et al., 2019), one percentages (Caplandies et al., 2017), two natural frequencies (Faasse et al., 2018a; Wilhelm et al., 2018), and two both percentages and natural frequencies (O’Connor et al., 1996; Webster et al., 2018b). Interpretation of the effects of statistical presentation was difficult in two studies. One employed percentages and natural frequencies separately across frames, potentially confounding the framing effect with altered perceptions of risk (Webster et al., 2018b). The other framed dizziness as either a positive or neutral consequence of treatment, but employed negative attribute framing (symptoms *occur* in 10 out of 100 people) in both cases (Wilhelm et al., 2018). Of the remaining, the largest framing effect was associated with verbal descriptors, followed by natural frequencies (Faasse et al., 2018a). As outlined in the subsequent section, inclusion of statistical information is advised to reduce the perception of side effect risk (Knapp et al., 2009). If the available data holds, then natural frequencies may be the optimal method of eliciting a framing effect.

An additional related factor concerns the number of side effects that patients are warned about. Evidence suggests that side effect warnings are better remembered, and nocebo side effects stronger, when fewer potential symptoms are listed (Colagiuri et al., 2012). Due to limitations on memory capacity, framing may therefore only be effective when warnings contain few side effects. This is difficult to deduce from the studies reviewed. Among those employing an attribute frame and reporting an effect, the number of listed side effects decreased as effect size increased. It is possible therefore, that limited memory for specific side effects weakens the framing effect, although more research is needed. One possible strategy to address this, would be to positively frame the overall likelihood of experiencing *any* side effects (as in, Fernandez et al., 2019), which may increase the salience of the positive frame, even with long lists of side effects.

### How Does Framing Influence the Perceived Absolute Risk of Side Effects?

An important ethical issue regarding the use of framing strategies to reduce the nocebo effect, concerns whether framing influences the perceived absolute likelihood of side effects. That is, if positive framing led to an underestimation of the absolute risk of side effects, then one could argue that informed consent was not actually being maintained because participants are not understanding the objective risk of side effects.

O’Connor et al. (1996) was the only study identified that examined perceived absolute risk of side effects. In their study, side effects were framed either negatively or positively according to their prevalence rates in the general population and participants were required to rate their perception of risk prior to treatment. Interestingly, both types of frames were associated with an increased perception of absolute risk relative to that outlined in the informed consent process. Importantly, estimates following the positive frame were closest to the objective statistical information – a finding consistent with studies risk perception following framing manipulations outside the nocebo effect (Jasper et al., 2001).

O’Connor et al. (1996) findings are, therefore, encouraging in terms of suggesting that positive framing does not compromise informed consent via a perceived underestimation of the absolute risk of side effects. However, given that it was the only study to test this in the context of nocebo side effects, we recommend that future studies investigating the effect of framing on side effects also incorporate an assessment of absolute risk perception as a matter of course so that researchers and clinicians can be confident that framing is not undermining informed consent.

### What Mechanisms Underlie the Framing Effect?

Expectancy is believed to play a key role in the development of the nocebo effect (e.g., Faasse and Petrie, 2013; Kirsch, 2018). Only two studies identified examined expectancy for side effects. One found that positive framing reduced side effect expectancy (O’Connor et al., 1996) and one found no such effect (Faasse et al., 2018a). However, despite being identified as a robust predictor of the nocebo effect (Webster et al., 2016), the precise psychological mechanisms through which expectancy gives rise to adverse side effects remains unresolved.

One line of evidence suggests that negative expectancies may elicit the nocebo effect by generating anticipatory anxiety (Benedetti et al., 2006; Colloca and Benedetti, 2007; Colagiuri and Quinn, 2018). This anticipatory anxiety has been proposed to generate increased attention toward internal bodily states and therefore adverse symptoms (Colloca and Benedetti, 2007). This is consistent with attentional theories outside of the placebo effect, such as chronic pain (e.g., Todd et al., 2015), where anxiety regarding the threat of pain leads to interpretation of pain as harmful, increasing the attentional focus on pain. Meta-analyses of attentional processes confirm biases toward sensory pain-related stimuli in patients with pain (Todd et al., 2018). Since, risk information can be processed by affective and cognitive systems (Slovic et al., 2004), we posit that positive framing attenuates anticipatory anxiety via the affective path, which in turn inhibits the nocebo effect by reducing the attention directed toward the aversive symptoms in question.

In terms of the studies identified, two provided data relevant to a possible role of anticipatory anxiety. Webster et al. (2018b) found that increased levels of anxiety were associated with elevated symptoms in the negative frame and Fernandez et al. (2019) reported a positive association between increased anxiety and side effects across groups. Faasse et al. (2018a) also measured anxiety, but this was the primary outcome for their placebo manipulation, not a possible mediator of framing. Webster et al.’s (2018b) results are consistent with evidence that side effect information delivered in PILs (typically negatively attribute framed) induce anxiety and fear in patients, particularly when many side effects are presented (Herber et al., 2014). Thus, as with assessing perceived absolute risk, we recommend that future studies examining framing and nocebo side effects explore expectancy, anxiety, and attentional biases in order to determine whether these processes underlie the framing effect.

### General Issues to Do With Research on the Nocebo Effect

In addition to the specific issues regarding framing and side effects described above, there are three general issues regarding nocebo research that should be considered when evaluating framing effects. First, only two studies reviewed included control groups to assess natural history (Faasse et al. (2018a) and no-frame instruction (Caplandies et al., 2017) conditions. Such control groups are important for determining the extent to which framing strategies influence side effects above and beyond processes such as the Hawthorne effect, including whether the framing effect generalizes to other symptoms. Second, only one study assessed how long the framing effect lasts (Faasse et al., 2018a). This is important, as nocebo effects are known to be easily instated, but resistant to extinction (Colagiuri et al., 2015; Colagiuri and Quinn, 2018; Faasse et al., 2018b). Third, inconsistent techniques were used for measuring side effects, including modified versions of the Generic Assessment of Side Effects Scale (GASE; Rief et al., 2011) and study-specific items (see Table 1). This lack of consistency makes cross-study comparisons difficult because the outcomes are not necessarily equivalent. Thus, as with nocebo effect research in general, we recommend that where possible future studies on framing and side effects incorporate appropriate control groups, evaluate the duration of any framing effects, develop and implement standardized measures to assess side effects, and consider potential methodological limitations when designing experiments (e.g., blinding, randomization and power: see Supplementary Material). Finally, preliminary evidence suggests that attribute and positive message framing may differentially impact side effect reporting, with the former decreasing, and the latter potentially increasing, side effect frequency. Further research is needed to disentangle these differences. Greater attention should also be paid to comparing the ability of these frame-types to uphold consent, ensuring that any intervention implemented is ethical.

## Conclusion

The handful of available studies suggests that positive valence framing of side effect warnings is a promising technique for reducing nocebo side effects, whilst maintaining informed consent. While the mechanisms are currently unknown, we propose that positive framing reduces anticipatory anxiety and subsequent attention to aversive symptoms, which then attenuates nocebo effects. Future research is, however, required to determine the optimal method of delivering such interventions, including the mode of delivery and statistical presentation of the side effects. Given that positive framing is relatively simple and cheap to implement, it has the potential to be a highly cost-effective technique for reducing the huge burden caused by nocebo effects.

## Author Contributions

All authors have contributed to the conception, review strategy, and interpretation of the results. KB drafted the initial version of the manuscript. KF, AG, SH, LC, LS, and BC contributed to refining the manuscript.

## Conflict of Interest Statement

The authors declare that the research was conducted in the absence of any commercial or financial relationships that could be construed as a potential conflict of interest.
