# Supplementary material for: Can Positive Framing Reduce Nocebo Side Effects? Current Evidence and Recommendation for Future Research
Source: Front Pharmacol. 2019 Mar 6;10:167. doi: 10.3389/fphar.2019.00167 (PMC6414427; doi:10.3389/fphar.2019.00167)
Supplement: Supplementary file 1 [file Presentation_1.pdf]

Methods

The objective was to provide an overview of research investigating the effect of framing on nocebo side effects, highlighting strengths and weaknesses. The ultimate goal was therefore to provide a theoretical foundation for emerging research in the field.

Search Strategy

A systematic search was conducted across several bibliographic databases (PsychINFO, Medline, Scopus and Pubmed), from inception to January 2019. Information regarding search terms can be found in Figure 1 of the main text. The reference sections and current citations of those studies identified as relevant were also examined. Grey literature was sought via OAISTER and OpenGrey. Protocol was established prior to conducting the search, but was not published or formally registered.

Selection Criteria

Eligible studies were published journal articles, printed in English, that collected data from adult participants (healthy or clinical populations). An experimental design must have been employed, with quantitative evidence reported. Case studies, interviews and reviews were excluded.

To be included, studies needed to have administered an ostensible treatment or medication (either active or placebo) and implemented a positive side effect framing manipulation and compared it with at least one other frame. Positive side effect framing manipulations were broadly defined as any in which information provided to participants was altered to accentuate the likelihood or significance of side effects in a positive light. In terms of dependent variables, studies must have measured the frequency and/or intensity of side effects and have reported appropriate descriptive statistics from which to calculate an effect size. To broaden the scope of the review, measurement could include the general experience of symptoms and/or those specifically attributed to treatment. Data collected from previously published, adapted, and study-specific questionnaires were considered. However, studies must have reported the frequency or intensity of those side effects framed during the consent process.

Study Coding

Citations (n = 255) were downloaded to Endnote (Endnote X8 Thomson Reuters, USA) where duplicates (n = 108) were removed. References were then exported to Rayyan (Ouzzani et al., 2016) where titles and abstracts were independently categorised according to inclusion and exclusion criteria by the lead author (K.B.) and an additional researcher not otherwise involved in the review (J.D., acknowledgements). At this stage, seven potentially relevant studies were identified. In a subsequent step, additional citations were sought by searching both the reference lists and published citations (via Google scholar) of the papers that appeared to fit the search criteria. This identified one further citation. The full text of the eight identified studies was subsequently assessed and two citations removed due to not adhering to search criteria. Both reviewers were in full agreement regarding included and excluded studies.

We note here that, while a criterion for inclusion was reporting of framed side effects, reporting was potentially ambiguous in one study (Fernandez et al., 2019). In this instance, eight side effects were mentioned during consent, but framing appeared to apply to any side effect experienced (wording: if you do experience a side effect, you might take this as a signal or reminder that the medication is absorbed and active in your body). We interpret this liberally as framing any side effect that might be experienced, thus legitimizing data extraction from the full GASE measure (Rief et al., 2011), which was not separated into those side effects listed and those not.

Data Extraction

For each included study, data was extracted pertaining to the following categories: sample characteristics, type of treatment administered, type of framing manipulation employed, communication method used when delivering framed information, statistical presentation of framed side effects, number of framed and non-framed side effects, outcome measure employed to assess side effects, whether side effects were measured generally or attributed to the treatment (for the largest effect only), measures used to assess expectancy for side effects, measures used to assess anxiety.

Statistical data was extracted for the primary outcome (frequency / intensity of framed side effects), and three secondary outcomes where available (frequency / intensity of non-framed side effects, effect of frame on side effect expectancy and effect of anxiety). In keeping with previous reviews concerning the effect of framing on health-related outcomes (O'Keefe and Jensen, 2007; 2009; Gallagher and Updegraff, 2012), Pearson's *r* was selected as the effect size. Where several results were reported for the primary outcome (e.g. frequency *and* intensity of side effect), we report the largest effect. We decided not to compute an averaged effect size because we did not perform quantitative synthesis and, given the review was the first of its type, we wanted to discuss the maximum potential impact of framing. We note, however, that overall combined effects are likely to be smaller. In some articles, Pearson's *r* was directly available. In other cases, means and standard deviations, Chi-square statistics or odds ratios were reported (for further information see Table 1). In these instances, the effect size was estimated from formulas suggested in the literature.

Quality Assessment

The intention of the review was not specifically to identify randomised controlled trials (RCTs), but any experimental evidence assessing the role of framing. In fact, only one study (Webster et al., 2018) reported being designed as an RCT. Placing weight on quality assessment designed for RCTs may therefore be considered stringent. For full transparency, we present data below based on the Cochrane Collaboration's Risk of Bias tool (Higgins et al., 2011), but allow the reader to form their own opinion regarding publication quality based on experiment type. Categories were assessed by the lead author (K.B.) and an additional researcher not otherwise involved in the review (J.D.). Discrepancies were resolved by a third researcher (B.C.).

Table 2: Risk of Bias Table (where the plus symbol represents ‘low risk of bias’, the minus symbol ‘high risk of bias’, and the question mark ‘unclear risk of bias’)

	Random sequence generation	Allocation concealment	Blinding: Participants	Blinding: Personnel	Blinding: Outcome assessment	Incomplete outcome data	Selective reporting	Power analysis
Fernandez et al. (2019)	?	+	+ [a]	+	+	+	? [b]	+
Faasse et al. (2018)	+	+	+	+	+	+	+	+
O’Connor et al. (1996)	?	?	+	+	+	+	− [c]	− [d]
Webster et al. (2018)	+	+	+	+	+	+	+	+
Wilhelm et al. (2018)	+	+	+	+	+	+	? [e]	−
Caplandies et al. (2017)	+	?	+	−	+	+	+	−

[a] Two methods of participant blinding were typical of the papers reviewed. In the first, participants were told that *k* groups existed, that they differed, and that they would be randomly assigned to one (O'Connor et al., 1996; Webster et al., 2018). In the second, participants were randomly assigned to an experimental condition, but were unaware others existed (Caplandies et al., 2017; Faasse et al., 2018; Wilhelm et al., 2018; Fernandez et al., 2019).

[b] Six indices were calculated from the GASE (Rief et al., 2011). When investigating analgesia and adverse events two are omitted. It is unclear whether these omissions were pre-planned.

[c] Only statistically significant Chi-square values are provided for side effect frequency. Some descriptive statistics are presented in graphical format and can only be estimated approximately.

[d] While no power analysis is reported, this study contains the largest sample of those reviewed.

[e] Exploratory analysis is reported for one subscale of the BMQ (Weinman and Hankins, 1999) only. It is unclear whether additional tests were performed and omitted.

References

Caplandies, F.C., Colagiuri, B., Helfer, S.G., and Geers, A.L. (2017). Effect type but not attribute framing alters nocebo headaches in an experimental paradigm. *Psychol. Conscious.* 4**,** 259-273. doi: 10.1037/cns0000130.

Faasse, K., Parkes, B., Kearney, J., and Petrie, K.J. (2018). The Influence of Social Modeling, Gender, and Empathy on Treatment Side Effects. *Ann. Behav. Med.* 52**,** 560-570. doi: 10.1093/abm/kax025.

Fernandez, A., Kirsch, I., Noël, L., Rodondi, P.Y., Kaptchuk, T.J., Suter, M.R., et al. (2019). A test of positive suggestions about side effects as a way of enhancing the analgesic response to NSAIDs. *PLoS ONE* 14**:**e0209851. doi: 10.1371/journal.pone.0209851.

Gallagher, K.M., and Updegraff, J.A. (2012). Health Message Framing Effects on Attitudes, Intentions, and Behavior: A Meta-analytic Review. *Ann. Behav. Med.* 43**,** 101-116. doi: 10.1007/s12160-011-9308-7.

Higgins, J.P.T., Altman, D.G., Gøtzsche, P.C., Jüni, P., Moher, D., Oxman, A.D., et al. (2011). The Cochrane Collaboration's tool for assessing risk of bias in randomised trials. *BMJ (Clinical research ed.)* 343**,** d5928-d5928. doi: 10.1136/bmj.d5928.

O'Connor, A.M., Pennie, R.A., and Dales, R.E. (1996). Framing effects on expectations, decisions, and side effects experienced: the case of influenza immunization. *J. Clin. Epidemiol.* 49**,** 1271-1276.

O'Keefe, D.J., and Jensen, J.D. (2007). The Relative Persuasiveness of Gain-Framed Loss-Framed Messages for Encouraging Disease Prevention Behaviors: A Meta-Analytic Review. *J. Health Commun.* 12**,** 623-644. doi: 10.1080/10810730701615198.

O'Keefe, D.J., and Jensen, J.D. (2009). The Relative Persuasiveness of Gain-Framed and Loss-Framed Messages for Encouraging Disease Detection Behaviors: A Meta-Analytic Review. *J. Commun.* 59**,** 296-316. doi: 10.1111/j.1460-2466.2009.01417.x.

Ouzzani, M., Hammady, H., Fedorowicz, Z., and Elmagarmid, A. (2016). Rayyan—a web and mobile app for systematic reviews. *Systematic Rev.* 5**,** 210. doi: 10.1186/s13643-016-0384-4.

Rief, W., Barsky, A.J., Glombiewski, J.A., Nestoriuc, Y., Glaesmer, H., and Braehler, E. (2011). Assessing general side effects in clinical trials: reference data from the general population. *Pharmacoepidemiol Drug Saf.* 20**,** 405-415. doi: 10.1002/pds.2067.

Webster, R.K., Weinman, J., and Rubin, G.J. (2018). Positively Framed Risk Information in Patient Information Leaflets Reduces Side Effect Reporting: A Double-Blind Randomized Controlled Trial. *Ann. Behav. Med.* 52*:*kax064. doi: 10.1093/abm/kax064.

Weinman, J., and Hankins, M. (1999). The beliefs about medicines questionnaire: The development and evaluation of a new method for assessing the cognitive representation of medication AU - Horne, Robert. *Psychology & Health***,** 1-24. doi: 10.1080/08870449908407311.

Wilhelm, M., Rief, W., and Doering, B.K. (2018). Decreasing the Burden of Side Effects Through Positive Message Framing: an Experimental Proof-of-Concept Study. *Int. J. Behav. Med.* 25**,** 381-389. doi: 10.1007/s12529-018-9726-z.
